# Modulation of the Endocannabinoids N-Arachidonoylethanolamine (AEA) and 2-Arachidonoylglycerol (2-AG) on Executive Functions in Humans

**DOI:** 10.1371/journal.pone.0066387

**Published:** 2013-06-19

**Authors:** Ana B. Fagundo, Rafael de la Torre, Susana Jiménez-Murcia, Zaida Agüera, Antoni Pastor, Felipe F. Casanueva, Roser Granero, Rosa Baños, Cristina Botella, Amparo del Pino-Gutierrez, Jose M. Fernández-Real, Jose C. Fernández-García, Gema Frühbeck, Javier Gómez-Ambrosi, José M. Menchón, Inés Moragrega, Roser Rodríguez, Salomé Tárrega, Francisco J. Tinahones, Fernando Fernández-Aranda

**Affiliations:** 1 Department of Psychiatry, University Hospital of Bellvitge-IDIBELL, Barcelona, Spain; 2 CIBER Fisiopatología Obesidad y Nutrición (CIBERObn), Instituto Salud Carlos III, Barcelona, Spain; 3 Human Pharmacology and Clinical Neurosciences Research Group, Neuroscience Research Program, IMIM (Hospital del Mar Medical Research Institute), Barcelona, Spain; 4 Department of Clinical Sciences, School of Medicine, University of Barcelona, Barcelona, Spain; 5 Department of Pharmacology, School of Medicine, Universitat Autònoma de Barcelona, Barcelona, Spain; 6 Endocrine Division, Complejo Hospitalario U. de Santiago, Santiago de Compostela University, Santiago de Compostela, Spain; 7 Departament de Psicobiologia i Metodologia, Universitat Autònoma de Barcelona, Barcelona, Spain; 8 Department of Personality, Evaluation and Psychological Treatment of the University of Valencia, Valencia, Spain; 9 Department of Basic Psychology, Clinic and Psychobiology of the University Jaume I, Castelló, Spain; 10 Nursing Department of Public Health, Maternal and Child Health the Nursing School of the University of Barcelona, Barcelona, Spain; 11 Department of Diabetes, Endocrinology and Nutrition, Institut d’Investigació Biomèdica de Girona (IdlBGi) Hospital Dr Josep Trueta, Girona, Spain; 12 Department of Endocrinology and Nutrition, Hospital Clínico Universitario Virgen de Victoria, Málaga, Spain; 13 Department of Endocrinology and Nutrition, Clínica Universidad de Navarra, University of Navarra, Pamplona, Spain; 14 CIBER Salud Mental (CIBERsam), Instituto Salud Carlos III, Barcelona, Spain; Nathan Kline Institute for Psychiatric Research and New York School of Medicine, United States of America

## Abstract

Animal studies point to an implication of the endocannabinoid system on executive functions. In humans, several studies have suggested an association between acute or chronic use of exogenous cannabinoids (Δ9-tetrahydrocannabinol) and executive impairments. However, to date, no published reports establish the relationship between endocannabinoids, as biomarkers of the cannabinoid neurotransmission system, and executive functioning in humans. The aim of the present study was to explore the association between circulating levels of plasma endocannabinoids N-arachidonoylethanolamine (AEA) and 2-Arachidonoylglycerol (2-AG) and executive functions (decision making, response inhibition and cognitive flexibility) in healthy subjects. One hundred and fifty seven subjects were included and assessed with the Wisconsin Card Sorting Test; Stroop Color and Word Test; and Iowa Gambling Task. All participants were female, aged between 18 and 60 years and spoke Spanish as their first language. Results showed a negative correlation between 2-AG and cognitive flexibility performance (r = −.37; p<.05). A positive correlation was found between AEA concentrations and both cognitive flexibility (r = .59; p<.05) and decision making performance (r = .23; P<.05). There was no significant correlation between either 2-AG (r = −.17) or AEA (r = −.08) concentrations and inhibition response. These results show, in humans, a relevant modulation of the endocannabinoid system on prefrontal-dependent cognitive functioning. The present study might have significant implications for the underlying executive alterations described in some psychiatric disorders currently associated with endocannabinoids deregulation (namely drug abuse/dependence, depression, obesity and eating disorders). Understanding the neurobiology of their dysexecutive profile might certainly contribute to the development of new treatments and pharmacological approaches.

## Introduction

Cannabis has been used for thousands of years and has long been associated with effects on cognitive and emotional processes. Investigation over the last decades has revealed that the effects of cannabinoids are mediated by their action on the endocannabinoid system. The endocannabinoid system comprises two receptors (CB1 and CB2) found predominately on presynaptic terminals of glutamatergic and GABAergic neurons [1]. The cannabinoid receptor 1 (CB1) is the most widely expressed G-protein coupled receptor in the brain and is associated with the majority of central effects of the cannabinoids [2]. Endogenous cannabinoid ligands (endocannabinoids) bind to cannabinoid receptors, and up to now, the arachidonate-derived lipid molecules N-arachidonoylethanolamine (anandamide; AEA) [3] and 2-arachidonoylglycerol (2-AG) [4] are the best studied and are considered as retrograde messengers in the brain.

The biosynthesis of endocannabinoids in the brain has been reviewed by Di Marzo [5]. Unlike the typical neurotransmitters, endocannabinoids are stored in the membrane as phospholipid precursors and released “on demand” by the elevation of intracellular Ca2+, membrane depolarization, or stimulation of metabotropic receptors. The endocannabinoids AEA and 2-AG are biosynthesized from different membrane phospholipid families, both esterified with arachidonic acid. For example, N-arachidonoylethanolamide, AEA is produced from N-arachidonoylphosphatidylethanolamines (NArPE). Several possible biosynthetic routes for the formation of AEA have been suggested with multiple enzymes implicated: the N-acylphosphatidylethanolamine specific phospholipase D (NAPE-PLD), the α,β-Hydrolase-4 (ABHD4), the glycerophosphodiesterase-1 (GDE1), a soluble phospholipase A2, an unidentified phospholipase C, and phosphatases. These biosynthetic pathways may be able to substitute one another as mice lacking NAPE-PLD do not show decreased AEA [6]. AEA is generally degraded by the fatty acid amide hydrolase (FAAH) enzyme [7]. Additionally, AEA can be degraded by two other enzymes, FAAH-2 and N-acylethanolamine acid amidase [8]. In contrast, the biosynthetic precursors for 2-AG, the sn-1-acyl-2-arachidonoylglycerols (AArG) are mostly produced by phospholipase Cβ (PLCβ) acting on membrane phosphatidylinositols, and then converted to 2-AG by the action of either of two isoforms of the same enzyme, the sn-1-diacylglycerol lipases α and β (DAGLα and DAGLβ) [9]. 2-AG is largely degraded by the monoacylglycerol lipase (MAGL) [7].

A network of interactions between the biosynthetic pathways of the two main brain endocannabinoids AEA and 2-AG has been suggested [5]: (i) the formation of their phospholipid precursors is dependent on the pool of arachidonic acid available; (ii) the degradation of AEA and 2-AG releases in both cases free arachidonic acid which can be rapidly re-esterified into phospholipids; (iii) in brain, 2-AG levels are approximately 200 times higher than AEA and the hydrolysis of 2-AG by MAGL contributes to determining free arachidonic acid levels, unlike anandamide hydrolysis [10].

The endocannabinoid system has been implicated in different behaviors, including food intake [11], the reinforcing characteristics of drugs of abuse [12] and cognitive processing [13]–[15]. The role of the endocannabinoid system in memory functioning has been widely studied [13], [14], apparently because of the high density of CB1 receptors in the hippocampus [16]. However, the endocannabinoid system appears to be implicated in other prefrontal-mediated cognitive functions such as the executive functions [17]. A major role of the endocannabinoid system in prefrontal activity was originally suggested by the elevated number of CB1 in this cerebral region, observed in both animals [18], [19] and humans [20]. Additionally, the endocannabinoids AEA and 2-AG are also found in this brain area [21], as well as the fatty acid amide hydrolase (FAAH) and monoacylglycerol Lipase (MAGL) [22], the enzymes accountable for AEA and 2-AG degradation [23], [24].

The effects of the endocannabinoid system on executive functioning have been extensively studied in animal models. In vitro experiments have indicated a clear role of endocannabinoids on behavioral flexibility, whereby reduced levels of 2-AG in the hippocampus resulted in poor flexibility [25], [26]. In addition, some animal studies suggested that endocannabinoids have a negative impact on set-shifting and cognitive flexibility, and that the use of antagonists of CB1 receptors can improve such executive functions [27]. Upregulation of the CB1 receptor, mainly in the prefrontal cortex, has also been associated with cognitive flexibility alterations in rats, assessed with attentional set shifting paradigms (an equivalent to the human Wisconsin card sorting test) [27]–[29] and olfactory go/no-go discrimination task [30]. Although the underling mechanisms remain ambiguous, it has been suggested that interactions with dopaminergic, GABAergic and glutamatergic transmission might be implicated [27], [29], [30].

In humans, several studies have suggested that acute consumption or administration of exogenous cannabinoid compounds (namely Δ9-tetrahydrocannabinol-THC) is associated with executive impairments. Indeed, acute use of THC in healthy controls is associated with alterations in response inhibition [31], decision making [32], and flexibility [33]. Acute administration of low doses of THC also modulates the cerebral inhibition response circuits (namely right inferior frontal cortex, anterior cingulate gyrus and posterior cingulated cortex) during a response inhibition task [34]. In the same line, after the acute administration of THC a consistent neural hyperactivity was observed on the prefrontal and anterior cingulated cortex [35]–[37], corroborating the hypothesis of the role of cannabinoids on frontal-mediated cognitive functions.

Chronic cannabis use has also been associated with executive functions deficits. Studies examining the degree of inhibitory control during a Stroop task concluded that cannabis users produced more errors of commission (failing to inhibit appropriately) than drug-free subjects and also showed an altered pattern of brain activation (namely reduced left anterior cingulate, bilateral dorsolateral prefrontal cortex, and right ventromedial prefrontal cortex activation) [38], [39]. Furthermore, dysfunctions in decision making (assessed with the Iowa Gambling Task) associated with reduced cortical activation, were observed in chronic cannabis users compared with non-drug users [40]. These results raise exciting questions about a plausible role of the endocannabinoid system on prefrontal-dependent cognitive functions in humans. However, to date, no published reports establish the impact of the endocannabinoid system on executive functioning, such as capacity of inhibition response, impulsivity, or decision making in humans.

In this study, we explored the relationship between circulating levels of plasma endocannabinoids (AEA and 2-AG) and executive functions (decision making, response inhibition, and cognitive flexibility) in healthy subjects in order to determine the plausible role of the endocannabinoid system on prefrontal-depended cognitive functioning. Hence, we used three neuropsychological tasks (Wisconsin Card Sorting Test; Stroop Color and Word Test; and Iowa Gambling Task) known to be mediated by prefrontal and orbitofrontal cortex functioning [17].

## Methods

### Sample

Seven centers, all involved in the CIBERobn Spanish Research Network, participated: the Eating Disorders Unit (Department of Psychiatry, University Hospital of Bellvitge-IDIBELL, Barcelona), the Department of Endocrinology at the University Hospital of Santiago (Santiago de Compostela); the Department of Diabetes, Endocrinology and Nutrition (Clinic University Hospital Virgen de Victoria, Málaga); the Department of Endocrinology (University of Navarra, Pamplona); the Diabetes, Endocrinology and Nutrition Department, Biomedical Research Institute of Girona (IdIBGi-Doctor Josep Trueta Hospital, Girona); the IMIM (Hospital del Mar Medical Research Institute, Barcelona); the Department of Basic Psychology, Clinic and Psychobiology (University Jaume I, Castelló). Enrolment in the study was between January 2010 and September 2012.

One hundred and fifty seven subjects (n = 157) were included. All participants were female, aged between 18 and 60 years, and spoke Spanish as their first language. Participants were recruited through several sources, including word-of-mouth and advertisements in the local university. The lifetime history of health or mental illnesses profile was based on the general health questionnaire (GHQ)-28. Prior to assessment, subjects were specifically asked about lifetime or current presence of drug or alcohol abuse or dependence (including cannabis abuse/dependence). Sociodemographic characteristics are presented in Table 1.

**Table 1:** Sociodemographic variables.

Age (years); *mean (SD)*	25.6 (7.8)
Education level; *%*	
* Primary*	6.7
* Secondary*	61.1
* University*	32.2
Employment status; *%*	
* Employed*	25.9
* Unemployed*	7.5
* Student*	47.6
* Student+Employed*	19.0
Civil status; *%*	
* Single*	72.8
* Married - in couple*	24.5
* Divorced - separated*	2.7
Tobacco use; *%*	
* Yes*	31.1
* Number of cigarettes-day; mean (SD)*	2.3 (4.6)

SD: standard deviation.

Exclusion criteria were: (1) Individuals who have suffered a lifetime disorder of the Axis I mental disorders; (2) History of chronic medical illness or neurological condition that might affect cognitive function; (3) Head trauma with loss of consciousness for more than 2 min, learning disability, or mental retardation; (4) Use of psychoactive medication or drugs; (5) Being male; and (6) Age under 18 or over 60 (to discard neuropsychological deficits associated with age).

All participants were informed about the research procedures and gave informed consent in writing. Procedures were approved by the Ethical Committee of each of the aforementioned institutions.

### Neuropsychological Assessment

As described in a previous study [41], all participants underwent a comprehensive neuropsychological and clinical assessment. The neuropsychological tests were selected to cover various aspects of executive functions including decision making, response inhibition, strategic planning and cognitive flexibility and were administered by a trained psychologist in a single session and in a randomized order. All participants were assessed with the following neuropsychological tests: (a) The Wisconsin Card Sorting Test (WCST) [42], (b) The Stroop Color and Word Test (SCWT) [43] and (c) The Iowa Gambling Task (IGT) [44]. These tests are well standardized and clear application norms are included in the manual, which guarantee the equivalence between administrators. For two tests (IGT and WCST), a computerized version was employed, with also help to avoid differences in correction between administrators. The protocol requires that the administrator remain unobtrusively present while the administration is taking place. However, before starting both, the IGT and WCST, the respondent was told to refrain from asking any questions until the completion of the test.

#### (a) Wisconsin card sorting test

The WCST is a classical measure of cognitive flexibility, conceptualized as the capacity to shift among stimuli. Subjects have to match a target card by color, number, or shape with one of four category cards and the classification rule is unpredictably changing. The test ends when the participant has completed 6 categories or 128 trials. The main outcome variable is the number of categories completed and higher scores indicate better cognitive flexibility and conceptualization. We also considered the number of errors and the number of cards used until the first category was successfully completed (initial conceptualization), as both variables are considered predictors of WCST results and mental set flexibility [45].

#### (b) Stroop color and word test

This paper and pencil test is a measure of inhibition response and interference control skills. Participants have 45 seconds to read as many words as possible in the first page (color words printed in black ink) and name the ink in pages 2 (“Xs” printed in color) and 3 (names of colors printed in an incongruent color). The main outcome variable is the “interference score” and higher scores in this variable indicate better capacity of inhibition response.

#### (c) Iowa gambling task

This task evaluates decision-making, risk and reward and punishment value. The subject has to select 100 cards from four decks (A, B, C and D). After each card selection an output is given: gain or a gain and loss of money. For decks A and B the final loss is higher than the final gain, while decks C and D are advantageous because the punishments are smaller. The outcome variable is the Total Score, with higher results point to better performance and higher capacity of decision making.

### Endocannabinoid Quantification Methods

Samples were always collected from subjects between 8 and 9 am after a fast of at least 12 hour duration. Blood obtained from human volunteers was centrifuged at 3500 rpm at 4°C for 15–20 min. Plasma aliquots were stored at −80°C until analysis. The endocannabinoid quantification was done with slight modifications of a previously described methodology of endocannabinoid analysis in brain tissue [46]. After the addition of the deuterated analogues (Cayman Chemical, USA) AEA-d4 (0.5 ng) and 2-AG-d5 (10 ng) to a 0.5 mL aliquot of plasma, endocannabinoids were extracted with a liquid-liquid extraction in tert-butyl-methyl-ether (Merck, Germany) and the extracts analyzed in a LC/MS-MS system (Agilent 6410, USA). The column used was a C8 (2.1×100 mm×1.8 µm particle size, Zorbax). The analysis was done in the multiple reaction monitoring mode (MRM) and the following precursor to product ion transitions was used: m/z 348→62 for AEA, m/z 352→66 for AEA-d4, m/z 379.2→287 for 2-AG and m/z 384→287 for 2-AG-d5. The quantification of AEA and 2-AG was done by isotopic dilution. Variations in accuracy and precision were <10% for the individual sample replicates. The limit of detection on column was 8 pg for AEA and 200 pg for 2-AG.

### Statistical Analysis

Analyses were carried out with SPSS 20 for Windows. Pearson’s correlation valued the linear association between endocannabinoids on cognitive outcomes. Next, multiple linear regressions measured the predictive power of endocannabinoids on cognitive measures. One model was adjusted for each cognitive outcome, entering simultaneously the two predictors (2-AG and AEA) and the covariates age, academic level, and tobacco use. The variables age, academic level, and tobacco use were also included as covariates into multivariate models since they achieved significant association with the outcomes (executive functions performance). R2 coefficients valued the global predictive validity of models.

## Results

Table 2 shows the descriptive values for endocannabinoids and cognitive outcomes. A negative correlation was found between 2-AG and cognitive flexibility performance. Specifically, there was a significant inverse correlation between 2-AG and both WCST total categories completed (r = −.37; p<.05) and WCST trials to complete the first category (r = −.38; p<.05). A significant positive correlation was found between AEA concentrations and both, cognitive flexibility [WCST total categories completed (r = .59; p<.05); WCST trials to complete the first category (r = .59; p<.05)] and decision making performance (IGT Total Score; r = .23; p<.05). We did not found any significant correlation between either 2-AG (r = −.17) or AEA (r = −.08) concentrations and Stroop performance.

**Table 2:** Descriptives (mean and standard deviation) for endocannabinoids and cognitive outcomes.

	*Mean*	*(SD)*
***Endocannabinoids***		
2-arachidonoyl glicerol (ng/mL)	1.60	(1.02)
Arachidonoyl ethanolamide (ng/mL)	0.55	(0.17)
***Cognitive variables***		
**STROOP**		
Interference	5.87	(7.71)
**WCST**		
Total errors	15.65	(13.51)
Categories completed	5.74	(1.18)
Trials first category	15.31	(18.14)
**IGT**		
Total score	17.09	(28.29)

Following a multiple regression model valuing the specific contribution of endocannabinoids on executive functions performance, 2-AG was significantly and inversely associated with Stroop interference (p = .05) and WCST performance (WCST total categories completed; p<.05; WCST trials to complete the first category; p<.05). 2-AG was not associated with the decision making performance (p = .28) (see Table 3). A significant contribution of AEA was also observed on WCST performance (WCST total categories completed; p<.001; WCST trials to complete the first category; p<.001). A trend toward significance was also found on IGT performance (total score; p = .09). AEA was not a predictor of inhibition response performance (p = .31) (see Table 3).

**Table 3:** Multiple regression models valuing the contribution of endocannabinoids on cognitive outcomes.

Criteria	2-arachidonoyl glycerol					Arachidonoylethanolamide					Summary		
	*p*	Beta	B	95% CI (B)		*p*	Beta	B	95% CI (B)		R^2^_T_	R^2^_A_ (p-value)	
**STROOP**													
Interference	**.05**	−.213	−1.40	−2.81	0.003	.31	−.109	−4.51	−13.2	4.22	.194	.041 (.135)	
**WCST**													
Total errors	.75	.039	0.52	−2.77	3.82	.89	.016	1.36	−18.8	21.5	.023	.001 (.951)	
Categories completed	**.005**	−.252	−112.8	−189.9	−35.7	**<.001**	.533	1,470.4	998.6	1,942.2	.519	.435 (**<.001**)	
Trials first category	.**005**	−.251	−111.2	−187.3	−35.0	**<.001**	.533	1,455.3	989.1	1,921.4	.521	.434 (**<.001**)	
**IGT**													
Total Score	.27	−.125	−3.42	−9.63	2.79	.09	.194	32.8	−5.22	70.8	.171	.069 (.053)	

Results adjusted by age, academic level and tobacco consumption. R^2^_T_: Total R^2^. R^2^_A_: R^2^ adjusted by covariates into the model. Bold: significant result.

## Discussion

The primary finding of this study is that the endocannabinoids AEA and 2-AG have a relevant and opposite role on the executive functioning in humans. According to our results, elevated levels of AEA are associated with improvement on decision making and cognitive flexibility performance, while elevated levels of 2-AG are associated with disruption of the cognitive flexibility and inhibition response capacities. These results demonstrate, in humans, the association between the endocannabinoid system and prefrontal-depended cognitive functions, and might have implications for the therapeutic use of drugs with cannabinoid activity.

Our results are in agreement with animal studies that demonstrate a clear and dose-related role of the endocannabinoid system in behavioral flexibility [27], [29]. Administration of high doses of CB1 receptor agonists increases impulsive behaviors, while the administration of low doses of CB1 antagonists improves set-shifting performance and reduces the number of impulsive responses [27], [47]. Genetic deletion of CB1 receptors also produces a significant impairment on reversal learning [15], [48], in an analogous way to that found after prefrontal cortex lesions [49]. A balance between novelty seeking and behavioral inhibition has also been found using mutant mice lacking the CB1 receptor either in cortical glutamatergic or GABAergic neurons [50] thus corroborating the found neural modulation of endocannabinoids on this executive function.

Our results are also supported by findings in chronic cannabis users, pointing to long-term and acute effects of the exogenous agonist of the CB1 receptors (such as derivates of the *Cannabis sativa*, namely THC) on executive functioning [51], [52]. Specifically, deficits in cognitive flexibility were reported in chronic cannabis users [53], [54] and seem to be persistent after 28 days of cannabis abstinence [54]. Impulsive behaviors also characterize the cognitive profile of cannabis users after both acute and chronic cannabis use [31], [33], [51], [55], and impairments on decision making are frequently reported in both recreational and chronic cannabis users [33], [56]. Similarly, it has been demonstrated that, compared with a placebo, subjects receiving acute administration of THC make more wrong decisions [33]. Finally, deregulation of the endocannabinoids system has been found in different psychiatric and neurologic disorders associated with executive dysfunctions, such as schizophrenia [57], Alzheimer disease [58], and Huntington disease [59]. Thus, our results point to a plausible implication of endocannabinoids on prefrontal cortex-depended dysfunction in such disorders.

As previously demonstrated in animal studies, the effects of the endocannabinoid system on executive functions might be explained by its action on some neurotransmitters, such as dopamine (DA), glutamate, or GABA, implicated in both prefrontal activity and executive functioning. In fact, endocannabinoids are retrograde messengers and are supposed to play a relevant role in synapses [60], [61]. Depending on the cerebral regions, the endocannabinoid system might produce activation or inhibition of neurotransmission, and consequently regulate the cognitive functions depending on these brain areas, including executive functions [62]–[64]. In this line, an enhanced prefrontal DA activity has been detected after administration of cannabinoids, modifying the prefrontal activity by elevating the release of DA in mesocortical neurons [65], [66]. It has also been demonstrated that the hyperactivity of prefrontal dopaminergic synapses induced by cannabinoid administration contributes to executive function deficits [67]. Additionally, CB1 receptor agonist suppresses the transmission of glutamate in the prefrontal cortex, producing impairments on executive functioning [68]. Specifically, a disruption in cognitive flexibility was observed after blocking the NMDA glutamate receptors within the prefrontal cortex [69], [70]. Furthermore, it was demonstrated that administration of a CB1 antagonist reduces the CB1 inhibition of glutamatergic activity in the PFC, which has been associated with activations in the prefrontal circuits implicated in executive functions [70]. Altogether, a plausible mechanism explaining the modulation of endocannabinoids on executive functions found in our study might by their actions on these neurotransmission systems.

Finally, the opposite effect produced by AEA and 2-AG plasma levels on executive functioning in humans might be explained because they derive from different biosynthetic pathways. However, as it has been suggested [5] it is extremely difficult to dissect AEA from 2-AG function. In addition to the interconnection of their biosynthetic routes with arachidonic acid metabolism, neurons have developed mechanisms through which anandamide and 2-AG can reciprocally control their biosynthesis, possibly as a way of fine-tuning endocannabinoid tone [5], [71], [72].

To our knowledge, this is the first study evaluating the role of endocannabinoids in executive functions in humans. However, these results must be interpreted in the context of some limitations. First, measures of intelligence quotient (IQ) were not considered, which might have influenced the executive performance. Nonetheless, years of education, as a cognitive level measure, has been considered in the statistical analysis. Second, only females were included in the study, thus the results are not applicable to males. Considering the sexual dimorphism observed in the endocannabinoid system [73], with males having higher levels of CB1 receptors [74] and females displaying a more efficient CB1 receptor activity [75], we decided to focus only on females. However, replication with a group including males should be considered. Additionally, cognitive flexibility, decision making, and inhibition response are complex cognitive functions supported by complex brain systems, and are hardly explained by only one task. Thus future studies should consider to include further decision-making, inhibition response, and cognitive flexibility tasks in order to better understand such executive variables. Furthermore, we use circulating levels of endocannabinoids, rather than central concentrations of endocannabinoids, as a measure of endocannabinoid system functioning. However, because of their elevated lipophilic characteristics, endocannabinoids easily cross the blood–brain barrier [76], it is therefore expected that the plasma levels of the endocannabinoids described in our study are almost certainly reflecting an equivalent central concentration of AEA and 2-AG. Finally, although a regression analysis was performed, our data are correlative in nature, meaning that they are indicative of associations between parameters and by no means demonstrate causality. Future studies with a longitudinal design should be performed in order to confirm the cause-effect relationships between endocannabinoids and executive functions in humans.

As a summary, this study demonstrates that, in humans, the endocannabinoid system plays an important role on prefrontal-dependent cognitive functioning, probably through mechanisms involving dopaminergic, cholinergic, GABAergic, and glutamatergic systems, as proved in animal models. The present study might have significant implications for the underlying executive alterations described in drug users [53], [54], obesity [41], and eating disorders [41], [77], [78], given the current body of evidence on the implication of the endocannabinoid system in these disorders [79]–[81]. Understanding the neurobiology of their dysexecutive profile might contribute to the development of new treatments and pharmacological approaches for these disorders.
